# Global, Regional, and National Burden of COPD Among Women from 1990 to 2021 and Projections to 2050: A Systematic Analysis for the Global Burden of Disease Study 2021

**DOI:** 10.5334/aogh.4883

**Published:** 2025-10-27

**Authors:** Shiyu Hu, Hao Chen, Jiaye Wang, Ye Zhang, Chengshui Chen, Ye Zhang, Wenyu Chen

**Affiliations:** 1 Department of Respiratory, The Affiliated Hospital of Jiaxing University, Jiaxing, Zhejiang 314000, PR China; 2 Zhejiang Chinese Medical University, Hangzhou, Zhejiang, 310053, PR China; 3 Department of General Medicine, The Affiliated Hospital of Jiaxing University, Jiaxing, Zhejiang 314000, PR China; 4 Department of Respiratory, The First Affiliated Hospital of Wenzhou Medical University, Wenzhou, Zhejiang, PR China

**Keywords:** chronic obstructive pulmonary disease, woman, disease burden, Global Burden of Disease 2021, Bayesian age-period-cohort

## Abstract

*Background:* With the increasing severity of the aging population and the rise in the number of female smokers, the prevalence of chronic obstructive pulmonary disease (COPD) among women continues to rise. Relevant research on what impact this situation exerts on the disease burden of COPD and its changing trends among women is currently lacking.

*Methods:* Data on female COPD burden were extracted from the Global Burden of Disease database for the years between 1990 and 2021. The trend of burden was evaluated by using percentage changes. Predicted trends for the years after 2021 were assessed by utilizing the Bayesian age-period-cohort (BAPC) model.

*Results:* In 2021, the global numbers of women with COPD were 112.7 million, with 1.6 million deaths and 34.7 million disability-adjusted life years (DALYs). The corresponding Age-standardized rate (ASRs) (per 100,000 population) were 2468.2, 34.1, and 750.6, respectively. Over the past 32 years, the age-standardized mortality rate (ASMR) and age-standardized DALY rate (ASDR) dropped by 36.9% and 35.0%, respectively. Among the five socio-demographic index (SDI) regions, in 2021, the low-middle SDI region had the highest age-standardized prevalence rate (ASPR), ASMR, and ASDR, at 2817.0 per 100,000 population, 70.7 per 100,000 population, and 1424.1 per 100,000 population, respectively. The BAPC analysis indicated that the number of women with COPD is expected to rise over the next 30 years, while ASPR, ASMR, and ASDR are projected to decline.

*Conclusions:* Although the ASPR, ASMR, and ASDR of female COPD patients have decreased from 1990 to 2021, the corresponding number of cases has been increasing, which reminds us that female COPD is indeed a public health issue that cannot be ignored. In the future, when formulating COPD prevention and control strategies, the special characteristics of female patients must be fully considered to reduce the disease burden of female COPD.

## Introduction

Chronic obstructive pulmonary disease (COPD) is a common, progressive respiratory disease characterized primarily by airflow limitation that is not fully reversible [1]. In 2019, COPD was the third leading cause of death globally. According to the latest Global Burden of Disease (GBD) data, COPD is currently the fourth leading cause of death worldwide, with an age-standardized death rate of 45.2 per 100,000 population [45.2 (95% UI: 40.7 to 49.8)] [2]. For a long time, the disease has been considered a male-dominated disease [3]. However, in recent years, with the gradual elevation in the life expectancy of women and the aging of the population, coupled with the rising number of female smokers, the disease burden of COPD in women has also become an undeniable public health issue [4]. According to the World Health Organization (WHO), an estimated 168 million men and 160 million women worldwide are affected by COPD [5]. The Global Initiative for Chronic Obstructive Lung Disease (GOLD) 2024 also mentioned that the prevalence of COPD in developed countries is almost equal between men and women [1, 6]. In some countries and regions, the incidence and mortality rates of women even surpass those of men [7–9]. A European Union project revealed that between 1994 and 2014, the age-standardized mortality rate (ASMR) for male COPD patients declined steadily by 2.56%, while that for female patients displayed only a 0.76% drop [10]. Consistent with these findings, the 2019 GBD study reported that among individuals aged 75 years and above, female cases outnumbered males, while for those aged 90 years and above, female deaths and disability-adjusted life years (DALYs) surpassed those of males [11]. Taken together, we believe that conducting a comprehensive in-depth analysis of the global burden of COPD disease in women is imminent, which can provide data support for female patients in the formulation of future COPD prevention and treatment strategies, thereby reducing the overall disease burden of female COPD patients.

## Materials and Methods

### Case definition and data sources

The GOLD definition of COPD was employed: the ratio of forced expiratory volume in one second (FEV1) to forced vital capacity (FVC) (FEV1/FVC), measured after bronchodilator use, was set to be <0.7 [2].

We utilized the Global Health Data Exchange Query Tool created by GBD collaborators [12] to collect existing data on female COPD. The GBD database is the most extensive and reliable disease burden database in the world, assessing the prevalence, mortality, and DALYs of 371 diseases and injuries in 204 countries and territories from 1990 to 2021, as well as 88 risk factors, and providing corresponding uncertainty intervals (UIs) [2]. We herein launched research on the latest updated data, collecting data on the number of women with COPD, deaths, DALYs, and their corresponding age-standardized rates (ASRs) globally, regionally, and nationally. In addition, we also collected data on global risk factors contributing to COPD-related DALY rates [13]. This work followed the Strengthening the Reporting of Observational Studies in Epidemiology (STROBE) reporting guidelines [14].

### Definition

#### SDI

The SDI is an all-encompassing indicator that comprises three components: per capita income, total fertility rate (under 25), and average years of schooling (at least 15). SDI serves as a measure of a nation’s overall social and economic development level, with the value ranging from 0 to 1. A score of 1 denotes the highest level of development, while a score of 0 indicates the lowest level [2]. Information on SDI’s regional division can be obtained from the website of the Institute for Health Metrics and Evaluation [15].

#### Smoking and tobacco

Specifically, we defined “smoking” as the current or past active use of combustible tobacco products (e.g., cigarettes, cigars), while “tobacco” refers more broadly to the use of any tobacco products, including smoking and smokeless forms (e.g., chewing tobacco).

### Statistical analysis

Considering the uncertainty in the original data source, data manipulation, measurement errors, and model selection, the Institute for Health Metrics and Evaluation’s Bayesian regression tool, DisMod-MR, was employed to analyze, model, and estimate indicators such as incidence, prevalence, mortality, and DALYs in this database. These indicators were standardized for the world population and reported as age-standardized prevalence rate (ASPR), ASMR, and age-standardized DALY rate (ASDR) per 100,000 population. All estimates were generated within 95% UIs, which encompassed all uncertainties arising from measurement errors, biases, and modeling [16]. Due to the age-standardized data in the GBD database, statistical analysis and plotting can be performed directly, and a 95% UI can be provided.

In this research, ASPR, ASMR, and ASDR served as primary indicators to describe the disease burden of female COPD. DALYs refer to the total number of healthy life years lost due to disease, starting from its onset and lasting until the individual dies or is still alive as of the evaluation date [17].

The analysis of the dynamic changes in COPD burden through percentage variations was applied to determine the time trends. A linear modeling technique was employed to estimate the 95% confidence intervals (95% CI) for the percentage variations. In this research, we employed the Bayesian agetimecohort (BAPC) model in conjunction with an integrated nested Laplace approximation to forecast the future trends of COPD cases, deaths, DALYs, and ASRs among female patients [18].

All statistical analyses and data visualizations were performed using R (version 4.4.2) and JD_GBDR (V2.37, Jingding Medical Technology Co., Ltd.).

## Results

### COPD in females: global level

**Figure 1:**
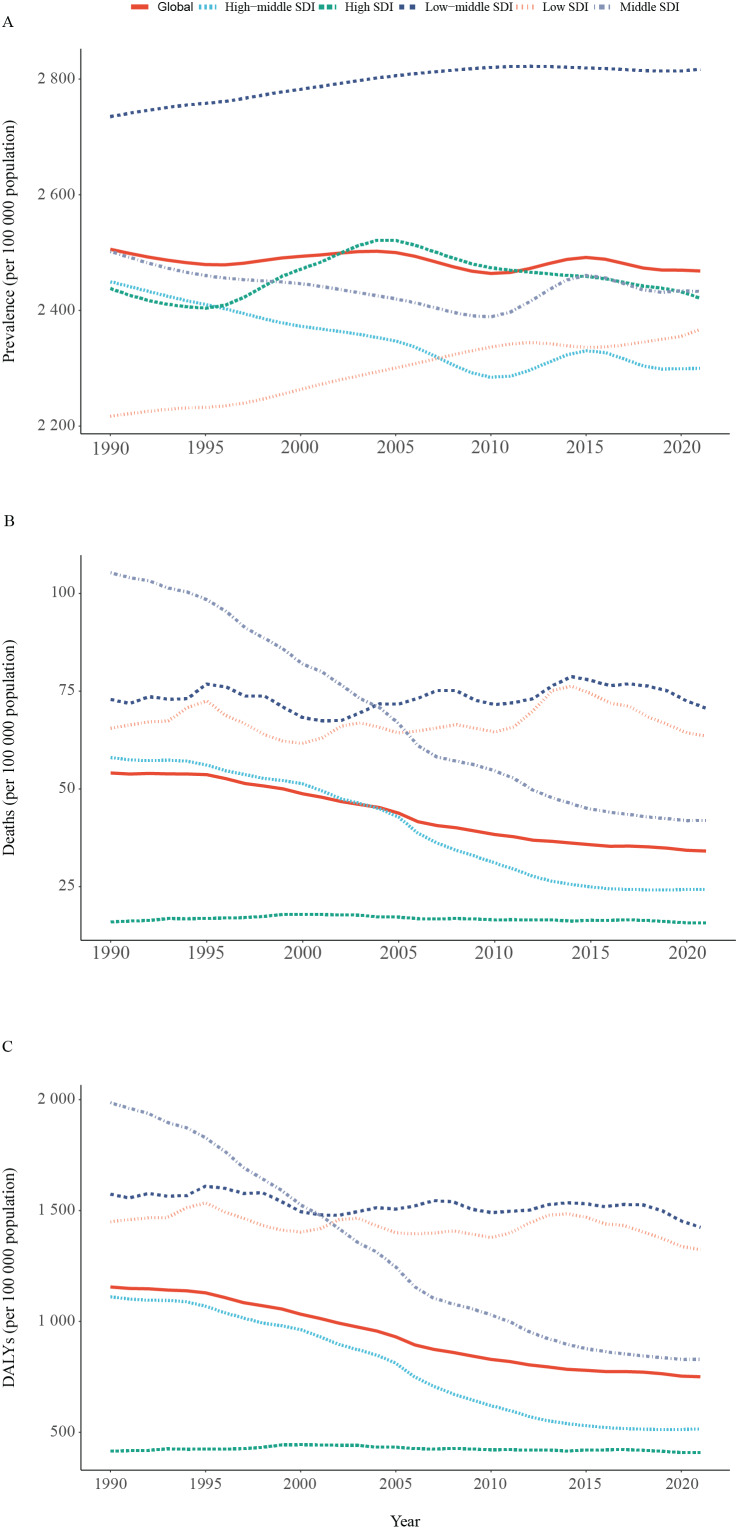
Temporal trends of ASPR **(A)**, ASMR **(B)**, and ASDR **(C)** for the burden of COPD among women, globally and by SDI from 1990 to 2021. ASPR, age-standardized prevalence rate; ASMR, age-standardized mortality rate; ASDR, age-standardized disability-adjusted life years (DALYs) rate; SDI, socio-demographic index (five categories: countries with a high, high-middle, middle, low-middle, or low socio-demographic index); COPD, chronic obstructive pulmonary disease.

In 2021, global cases of COPD in women numbered 112.7 million, with an ASPR of 2468.2 per 100,000 population for COPD in females. That same year, COPD mortality in women was 1.6 million, with an ASMR of 34.1 per 100,000 population, which reduced by 36.9% since 1990. In 2021, the number of female COPD-related DALYs globally was 34.7 million, with an ASDR of 750.6 DALYs per 100,000 population, exhibiting a decline of 35.0% since 1990 (Table 1 and Figure 1).

**Table 1:** Prevalent cases, deaths, and DALYs for COPD among females in 2021, and percentage change in ASRs per 100,000, by SDI and GBD region, from 1990 to 2021.

	PREVALENCE			DEATHS			DALYs		
	No. in millions (95% UI)	ASRs per 1000,000 (95% UI)	Percentage change in ASRs from 1990 to 2021	No. in thousands (95% UI)	ASRs per 1000,000 (95% UI)	Percentage change in ASRs from 1990 to 2021	No. in thousands (95% UI)	ASRs per 1000,000 (95% UI)	Percentage change in ASRs from 1990 to 2021
Global	112.7 (102.5 to 123.6)	2468.2 (2245 to 2704)	−1.5 (-3.4 to 0.4)	1599.5 (1355.1 to 1856.9)	34.1 (28.9 to 39.6)	−36.9 (−46.8 to −17.7)	34721.9 (30574.6 to 39278.8)	750.6 (662 to 848.2)	−35.0 (−43.5 to −18.4)
5 SDI region									
Low SDI	6.6 (6 to 7.2)	2367.1 (2148.5 to 2588.1)	6.8 (4.0 to 9.1)	123.9 (99.4 to 156.7)	63.5 (51.0 to 79.8)	−3.0 (−22.2 to 46.7)	3122.4 (2614.7 to 3815.9)	1323.8 (1102.2 to 1628.9)	−8.7 (−24.5 to 27.6)
Low-Middle SDI	20.8 (18.9 to 22.8)	2817.0 (2571.6 to 3076.7)	3.0 (1.1 to 4.7)	440.6 (345.7 to 523.7)	70.7 (55.6 to 84.0)	−3.0 (−22.9 to 60.6)	9892.3 (8154.5 to 11494.5)	1424.1 (1173.2 to 1655.2)	−9.5 (−25.5 to 38.1)
Middle SDI	33.4 (29.8 to 37.3)	2432.8 (2172.5 to 2713.4)	−2.8 (−5.9 to 0.2)	528.1 (419.9 to 650.7)	42.0 (33.2 to 51.7)	−60.1 (−69.4 to −45.2)	11096.0 (9351.0 to 13253.9)	829.3 (697.4 to 991.7)	−58.3 (−66.5 to −45.1)
High- middle SDI	24.4 (21.7 to 27.3)	2299.7 (2061.5 to 2565.8)	−6.1 (−9 to −3.4)	284.8 (223.7 to 351.1)	24.4 (19.2 to 30.1)	−58 (−68.3 to −42.2)	5732.9 (4841.0 to 6756.6)	515.6 (439.0 to 606.0)	−53.6 (−62.5 to −39.6)
High SDI	27.5 (25.4 to 29.5)	2421.2 (2261.6 to 2591.5)	−0.7 (−4.2 to 3.4)	221.3 (185.3 to 240.9)	15.8 (13.5 to 16.9)	−1.5 (−6.8 to 4.9)	4860.1 (4383.2 to 5227.0)	408.8 (374.9 to 437.8)	−1.5 (−4.8 to 2.5)
21 GBD region									
Andean Latin America	0.5 (0.4 to 0.5)	2470.9 (2199.3 to 2772.3)	1.5 (−9.4 to 13.5)	3.4 (2.5 to 4.3)	11.1 (8.2 to 14.2)	−30.9 (−47 to −11)	68.6 (54.2 to 83.7)	223.3 (175.5 to 273)	−29.1 (−42.5 to −12.7)
Australasia	0.4 (0.4 to 0.5)	1914.3 (1699.8 to 2151.5)	−17.6 (−26.9 to −7.3)	5.5 (4.5 to 6)	16.2 (13.7 to 17.7)	−4.5 (−12.6 to 1.8)	103.3 (91 to 111.7)	343 (306 to 370.1)	−15.3 (−20.8 to −10.7)
Caribbean	0.5 (0.5 to 0.6)	2542.6 (2303.8 to 2799.8)	17.1 (8.9 to 25.4)	4.6 (3.9 to 5.6)	15.2 (13 to 18.6)	8.3 (−5.4 to 27.7)	104.3 (89.2 to 125.3)	360.2 (307.1 to 435.5)	12.3 (0.4 to 30)
Central Asia	1 (0.9 to 1.2)	2336.4 (2114.1 to 2596.9)	−0.3 (−5.9 to 5.1)	5.7 (5.1 to 6.6)	14.4 (12.7 to 16.4)	−43.8 (−50.2 to −35.9)	158 (141.7 to 178.4)	362.5 (325.4 to 406.2)	−37.8 (−43.7 to −29.9)
Central Europe	2.8 (2.5 to 3.1)	2285.2 (2075 to 2531.3)	3 (−1.8 to 8.5)	14.9 (13.4 to 16.2)	10.2 (9.2 to 11.1)	−39.8 (−44.5 to −34.9)	371.1 (338.1 to 405.3)	289.8 (264.4 to 316.7)	−26.2 (−30.8 to −21.7)
Central Latin America	2.9 (2.6 to 3.2)	2023.7 (1792.5 to 2263.3)	−1.5 (−5.9 to 3)	29.4 (25.4 to 32.8)	22.5 (19.5 to 25.1)	−24.4 (−32 to −16.9)	576 (515.1 to 634.7)	434.9 (389 to 478.9)	−23.3 (−29.5 to −16.8)
Central Sub-Saharan Africa	0.6 (0.6 to 0.7)	1210.1 (1068.4 to 1369.1)	10.4 (4.3 to 17.7)	8.3 (4.9 to 16.8)	38.7 (22.8 to 80.6)	−13.8 (−38.2 to 21.8)	249.7 (165.2 to 422.6)	902.5 (593.7 to 1632.3)	−10.6 (−32.4 to 21.3)
East Asia	27.1 (24 to 30.7)	1513.2 (1329 to 1724.4)	−13.2 (−18.3 to −8.6)	552.8 (413.9 to 708.3)	52.3 (39 to 66.8)	−73 (−81.1 to −60.4)	10526.8 (8388.4 to 13073.4)	958.5 (767 to 1187.9)	−70.8 (−78 to −58.5)
Eastern Europe	4.2 (3.6 to 4.7)	2558.4 (2335.8 to 2798.4)	−14.5 (−18.3 to −11.4)	12.9 (11.6 to 14.2)	5.3 (4.7 to 5.8)	−74.8 (−76.8 to −72.4)	408.6 (368.2 to 451.5)	191.2 (172.6 to 211.2)	−58.9 (−61.6 to −56)
Eastern Sub-Saharan Africa	1.6 (1.4 to 1.8)	1350.6 (1188.6 to 1535.4)	3.8 (0.7 to 6.6)	15.4 (11 to 21.3)	22.4 (15.9 to 31.3)	−22 (−39.4 to 10.3)	504.1 (388.6 to 633.4)	562.3 (431.7 to 707.3)	−18.6 (−33.8 to 8.6)
High-income Asia Pacific	3.3 (2.8 to 3.9)	3286.6 (3112 to 3443.1)	−18.4 (−22.7 to −13.7)	13.3 (9.5 to 15.8)	3 (2.2 to 3.6)	−64.3 (−69.5 to −57.1)	303.4 (252.4 to 353.5)	98 (85.1 to 114.7)	−48.9 (−52.6 to −43.5)
High-income North America	11.7 (11 to 12.2)	1758.3 (1573.6 to 1951)	8.6 (2.4 to 16.5)	112.6 (95.3 to 121.4)	27.6 (23.7 to 29.5)	49.3 (41.4 to 53.9)	2579.2 (2340.7 to 2762.5)	700.8 (645.3 to 750.2)	24.4 (19.6 to 28.7)
North Africa and the Middle East	5.7 (5.1 to 6.3)	1503.3 (1318.5 to 1705.8)	20.6 (16.1 to 25.4)	35.8 (30.2 to 41.2)	20.8 (17.4 to 24.1)	–24.4 (–38.5 to 15.2)	1043 (924 to 1169.2)	499.4 (442.2 to 559.2)	−20.1 (−32.2 to 10.7)
Oceania	0.1 (0.1 to 0.1)	2174.2 (1967.6 to 2395.4)	−5.9 (−9 to −2.8)	2.9 (2.2 to 3.8)	109.9 (83.6 to 142)	–14.9 (–36 to 25.2)	78.2 (58.8 to 100.8)	2297.4 (1740.8 to 2924)	−16.4 (−37.1 to 20.9)
South Asia	23.8 (21.7 to 25.9)	2442.4 (2157.7 to 2750.2)	1.9 (0 to 3.5)	549.3 (430.9 to 669.9)	87.3 (68.6 to 106.5)	−6.3 (−28.4 to 65.9)	12363.2 (10084.8 to 14609.6)	1766.2 (1438.9 to 2092.8)	−11.7 (−29.4 to 40.7)
Southeast Asia	6.6 (5.8 to 7.4)	2530.1 (2254.1 to 2820)	−0.7 (−3.5 to 2.1)	79.2 (65.3 to 99.6)	27 (22.3 to 33.8)	−33.1 (−48.8 to 18)	1927.2 (1636.5 to 2314.6)	587.5 (498.4 to 705.4)	−31.4 (−45.9 to 10.1)
Southern Latin America	0.7 (0.6 to 0.8)	3215 (2928.3 to 3502.7)	0 (−10.2 to 12.2)	9.4 (8.2 to 10.1)	16.8 (14.8 to 18)	5.2 (−2 to 11.5)	167.2 (152.7 to 178.4)	325.6 (298.8 to 346.4)	−1.1 (–6.7 to 3.9)
Southern Sub-Saharan Africa	0.7 (0.6 to 0.8)	2020.5 (1785.2 to 2297.2)	1.2 (−2.1 to 4.4)	7 (6.1 to 7.8)	24.2 (21.3 to 27)	−5.4 (−38.1 to 21.3)	214.8 (193.5 to 240.4)	662.5 (596.2 to 741.9)	−3.4 (−28.1 to 11.9)
Tropical Latin America	3.4 (3 to 3.9)	1523.5 (1335.1 to 1701.7)	−4.2 (−7.8 to −0.3)	30 (25.8 to 32.5)	21 (18 to 22.6)	−33.4 (−38.5 to −29.1)	658.3 (600.2 to 704.1)	463.7 (423.3 to 495.7)	−27.9 (−32.2 to −24.2)
Western Europe	13.1 (11.8 to 14.3)	2045.7 (1802 to 2300)	3 (−0.2 to 6.5)	93.6 (76.8 to 102.8)	13.6 (11.5 to 14.7)	0.5 (−5.4 to 4.5)	1815.7 (1600.3 to 1967)	325.1 (292.2 to 350.9)	1.2 (−2 to 3.8)
Western Sub-Saharan Africa	2 (1.8 to 2.3)	1636.1 (1434 to 1845.6)	10.1 (6.7 to 13.4)	13.6 (10.8 to 17.1)	16.9 (13.8 to 20.9)	−17.9 (−35.9 to 20.1)	501.1 (418.1 to 602.1)	470.1 (396 to 561.5)	−12 (−27.5 to 15.8)

DALYs, disability-adjusted life years; COPD, chronic obstructive pulmonary disease; No., number; 95% UI, 95% uncertainty intervals; ASRs, age-standardized rates; SDI, socio-demographic index; GBD, Global Burden of Disease.

### COPD in females: SDI level

In 2021, in low-middle SDI regions, female COPD patients had the highest ASPR (2,817.0), ASMR (70.7) and ASDR (1424.1) (per 100,000 population), while those in high-middle SDI regions (2,299.7) had the lowest ASPR and in high SDI regions had the lowest ASMR (15.8) and ASDR (408.8) (Table 1).

From 1990 to 2021, there has been an upward trend in the prevalence of ASPR among female COPD patients in low-SDI and low-middle SDI regions [low SDI: 6.8 (95% CI: 4.0 to 9.1); low-middle SDI: 3.0 (95% CI: 1.1 to 4.7)]. In the high-middle SDI region, ASPR had a decline of 6.1% [−6.1 (95% CI: −9 to −3.4)] since 1990. From 1990 to 2021, there were clear downward trends in ASMR [middle SDI: −60.1(95% CI −69.4 to −45.2), high-middle SDI: −58.0(95% CI −68.3 to −42.2)] and ASDR [middle SDI: −58.3(95% CI −66.5 to −45.1), high-middle SDI: −53.6(95% CI −62.5 to −39.6)] for the middle SDI and high-middle SDI regions (Table 1 and Figure 1).

### COPD in females: regional level

**Figure 2:**
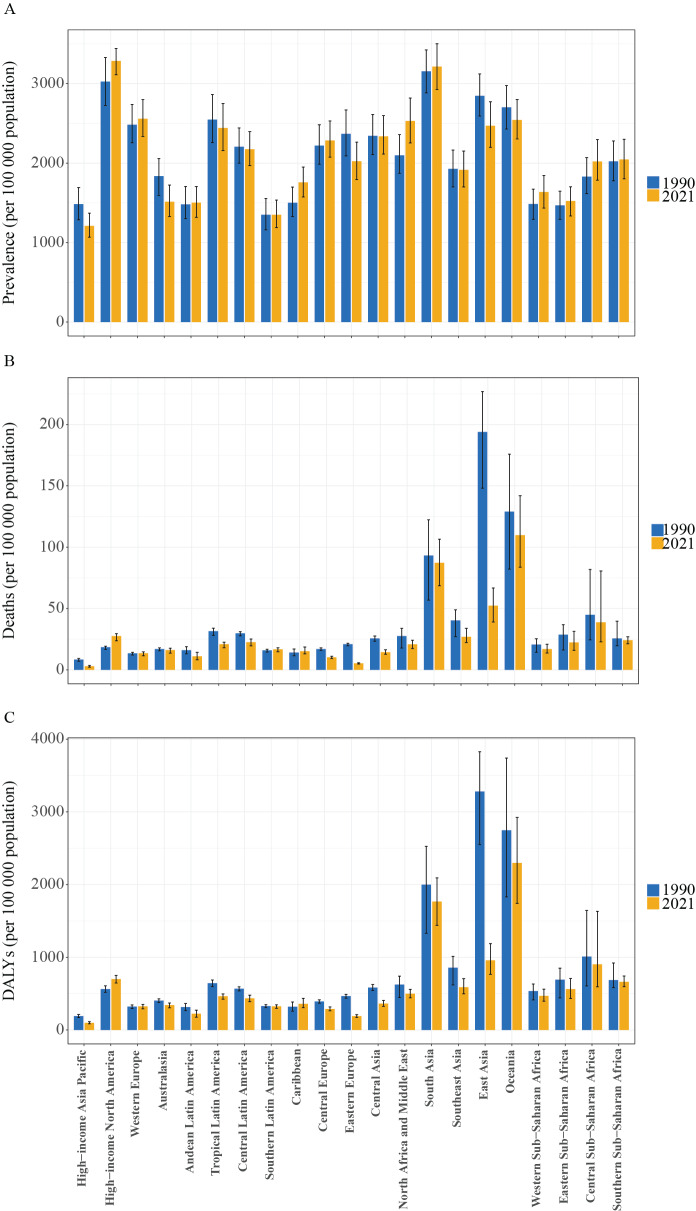
ASPR **(A)**, ASMR **(B)**, and ASDR **(C)** in 1990 and 2021 for the burden of COPD among women by 21 GBD regions. ASPR, age-standardized prevalence rate; ASMR, age-standardized mortality rate; ASDR, age-standardized disability-adjusted life years (DALYs) rate; COPD, chronic obstructive pulmonary disease; GBD, Global Burden of Disease Study.

In 2021, ASPRs (per 100,000) for high-income North America (3286.6), South Asia (3215.0), and Western Europe (2558.4) were the highest. ASMR and ASDR of female COPD patients were highest in Oceania, South Asia, and East Asia. The ASMRs in these regions were 109.9 per 100,000, 87.3 per 100,000, and 52.3 per 100,000, respectively, while the ASDRs were 2,297.4 per 100,000, 1,766.2 per 100,000, and 958.5 per 100,000, respectively (Table 1 and Figure 2).

Between 1990 and 2021, there was an upward trend in ASPR in the following regions: North Africa and the Middle East [20.6 (95% CI: 16.1 to 25.4)], the Caribbean [17.1 (95% CI: 8.9 to 25.4)], Central Sub-Saharan Africa [10.4 (95% CI: 4.3 to 17.7)], Western Sub-Saharan Africa [10.1 (95% CI: 6.7 to 13.4)], high-income North America [8.6 (95% CI: 2.4 to 16.5)], and Eastern Sub-Saharan Africa [3.8 (95% CI: 0.7 to 6.6)]. Among them, North Africa and the Middle East had the greatest uplifts, with a growth rate of 20.6%. In contrast, the ASMR in Eastern Europe decreased by 74.8%. During the same period, the ASMR grew by 49.3% in high-income North America [49.3 (95% CI: 41.4 to 53.9)], while ASMRs dropped in Eastern Europe [−74.8 (95% CI: −76.8 to −72.4)], marking the largest decline among the 21 regions. In terms of DALYs, ASDR were increasing in high-income North America [24.4 (95% CI: 19.6 to 28.7)] and the Caribbean [12.3 (95% CI: 0.4 to 30)] (Table 1 and Figure 2).

### COPD in females: national level

**Figure 3:**
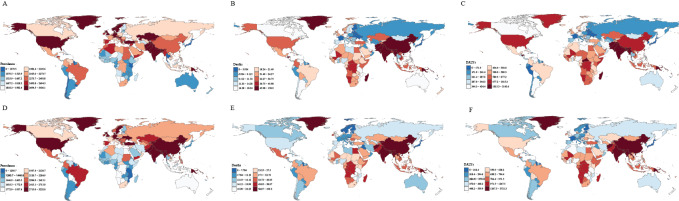
National ASPR **(A)**, ASMR **(B)**, and ASDR **(C)** in 2021. National ASPR **(D)**, ASMR **(E)**, and ASDR **(F)** in 1990 for overall female COPD. ASPR, age-standardized prevalence rate; ASMR, age-standardized mortality rate; ASDR, age-standardized disability-adjusted life years (DALYs) rate; COPD, chronic obstructive pulmonary disease.

In 2021, ASPRs for women with COPD among countries varied between 716.9 cases per 100,000 individuals and 3436.1 cases per 100,000 individuals, with the top three being the United States of America (3436.1 (95% UI: 3255.5 to 3594.3)), Bangladesh (3317.5 (95% UI: 2976.8 to 3707.8)), and the United Kingdom (3306.7 (95% UI: 2984.8 to 3617.5)). Singapore [716.9 (95% UI: 612.2 to 848.9)] had the lowest ASPR in 2021. In 2021, Papua New Guinea exhibited the highest ASMR [158.9 (95% UI: 116.9 to 212.1)] followed by Nepal [134.6 (95% UI: 90.3 to 181.4)] and India [93.9 (95% UI: 69.4 to 114.4)]. Kuwait [1.6 (95% UI: 1.2 to 1.8)] exhibited the lowest ASMR. In 2021, the ASDR for female COPD patients ranged from 72.0/100,000 to 4091.0/100,000, with the highest three countries being Papua New Guinea [3143.6 (95% UI: 2323 to 4091)], Nepal [2633.6 (95% UI: 1862.6 to 3425.4)], and India [1873 (95% UI: 1486.7 to 2226.8)], while the lowest countries were Singapore [72 (95% UI: 63.1 to 82.5)] (Table S1 and Figure 3).

From 1990 to 2021, the trends in ASPR among female COPD patients varied across countries. Morocco experienced the largest increase, with a rise of 39.8% [39.8 (95% CI: 27.7 to 54.1)], while Singapore showed the most significant decline at −36.2% [95% CI: −44.9 to −26.4]. During the same period, Norway recorded the largest increase in ASMR [233.7 (95% CI: 211.0 to 252.3)] and ASDR [118.1 (95% CI: 102.2 to 133.2)]. In contrast, Belarus had the greatest decline in ASMR [−91.6% (95% CI: −93.2 to −89.9)], and Singapore showed the largest reduction in ASDR [−80.8% (95% CI: −82.7 to −78.4)] (Table S1 and Figure 3).

### Age patterns

**Figure 4:**
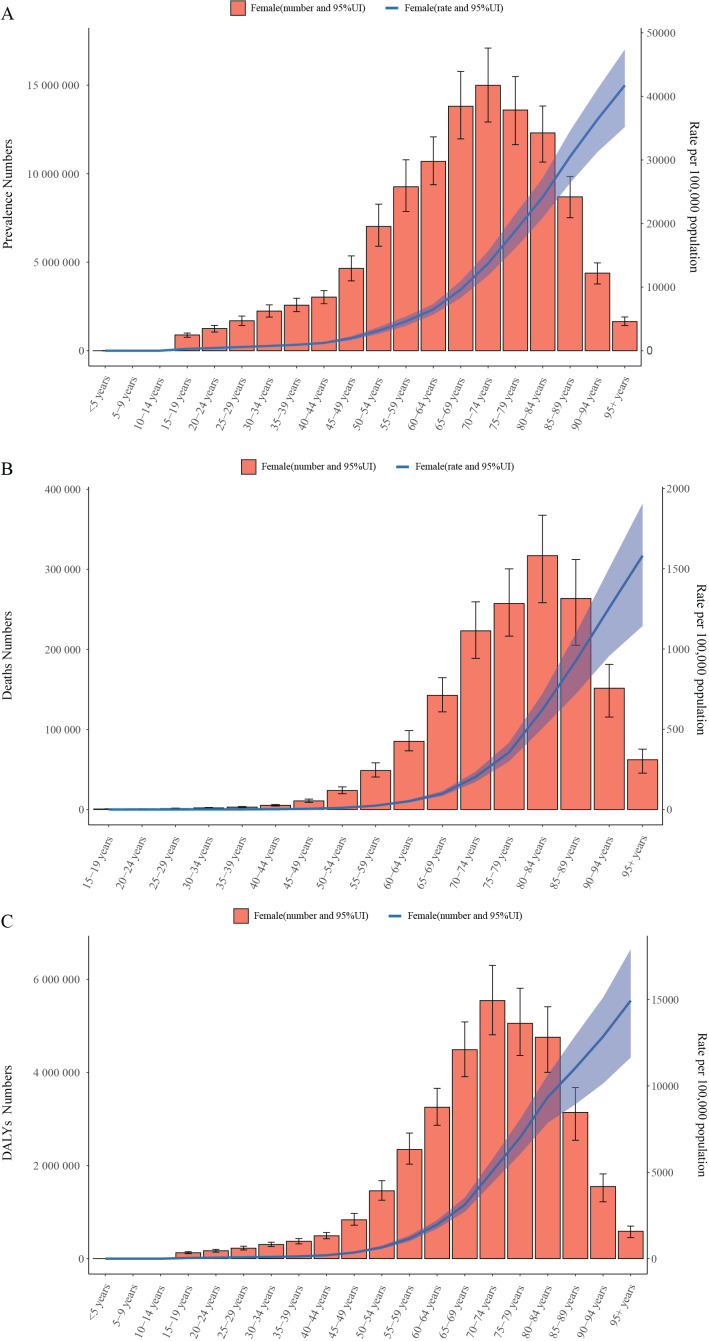
Numbers (bar plot) and ASRs (line plot) of prevalence **(A)**, deaths **(B)**, and DALYs **(C)** of COPD among women, by age in 2021. The blank vertical lines indicate prevalent cases with 95% UI for women. ASRs, age-standardized rates; DALYs, disability-adjusted life years; COPD, chronic obstructive pulmonary disease.

In 2021, the number of prevalent cases, deaths, and DALYs due to COPD in females exhibited a trend of increasing with age, followed by a decline. The number of prevalent cases and DALYs peaked in the 70–74 age group, reaching 15.0 million cases and 5,546,171 DALYs, respectively. Meanwhile, the number of mortality cases in females peaked in the 80–84 age group, with 317,114 deaths recorded in 2021. ASPR, ASMR, and ASDR all increased with age, reaching their highest levels in the 95+ age group. The values were 41.7 per 100,000 for ASPR, 1,581.3 per 100,000 for ASMR, and 14,939.3 per 100,000 for ASDR (Table S2 and Figure 4).

### Association with the SDI

**Figure 5:**
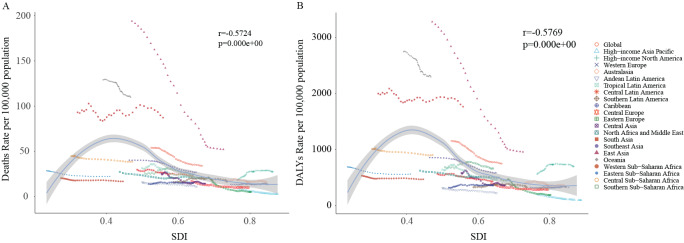
ASRs of female COPD in 21 GBD regions by SDI, 1990–2021. **(A)** ASMR; **(B)** ASDR. The blue line indicates an adaptive association fitted with adaptive Loess regression based on all data points. ASRs, age-standardized rates; GBD, Global Burden of Disease Study; SDI, socio-demographic index; COPD, chronic obstructive pulmonary disease; ASMR, age-standardized mortality rate; ASDR, age-standardized disability-adjusted life years (DALYs) rate.

At the SDI regional level, we used Spearman correlation and linear regression analysis to explore the relationship of SDI with ASMR and ASDR in female COPD patients. We found that from 1990 to 2021, there was an inverted V-shaped association of SDI with ASMR and ASDR in female COPD patients. As the SDI increased, the ASMR showed a trend of first elevating and then declining, reaching its peak at an SDI of 0.42. From 1990 to 2021, according to SDI, we discovered that ASMR levels in South Asia, Oceania, East Asia, and high-income North America were higher than expected. Compared with ASMR, ASDR also exhibited a similar trend (Figure 5).

### Prediction of global COPD among females

**Figure 6:**
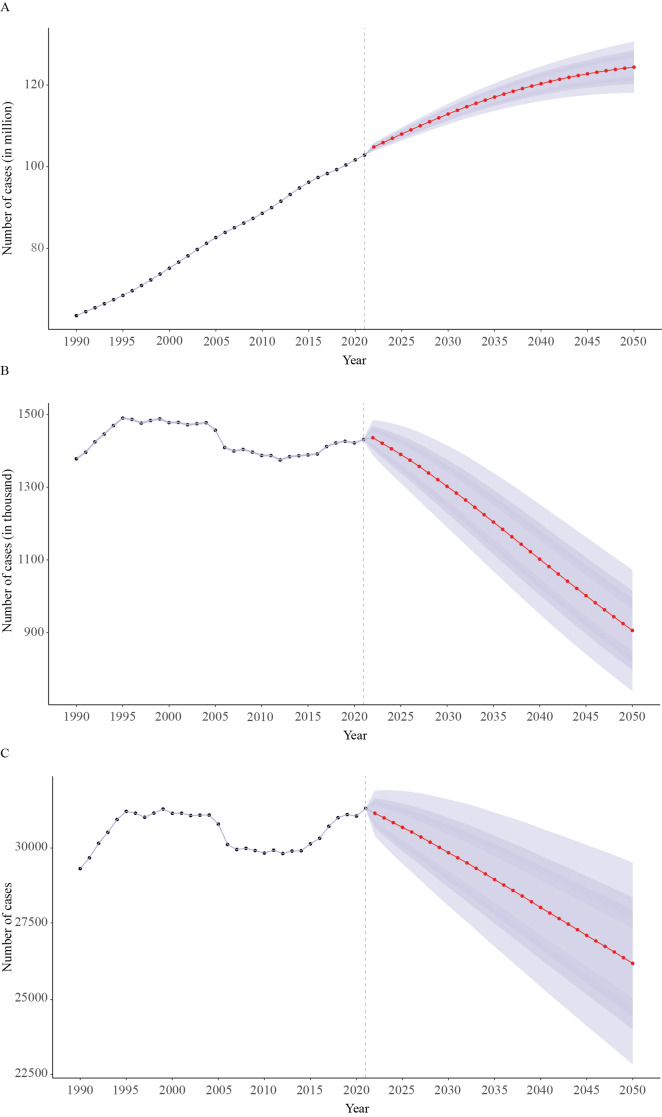
Global predictions of the numbers of prevalence **(A)**, deaths **(B)**, and DALYs **(C)** for female COPD from 2022 to 2050. DALYs, disability-adjusted life years; COPD, chronic obstructive pulmonary disease.

**Figure 7:**
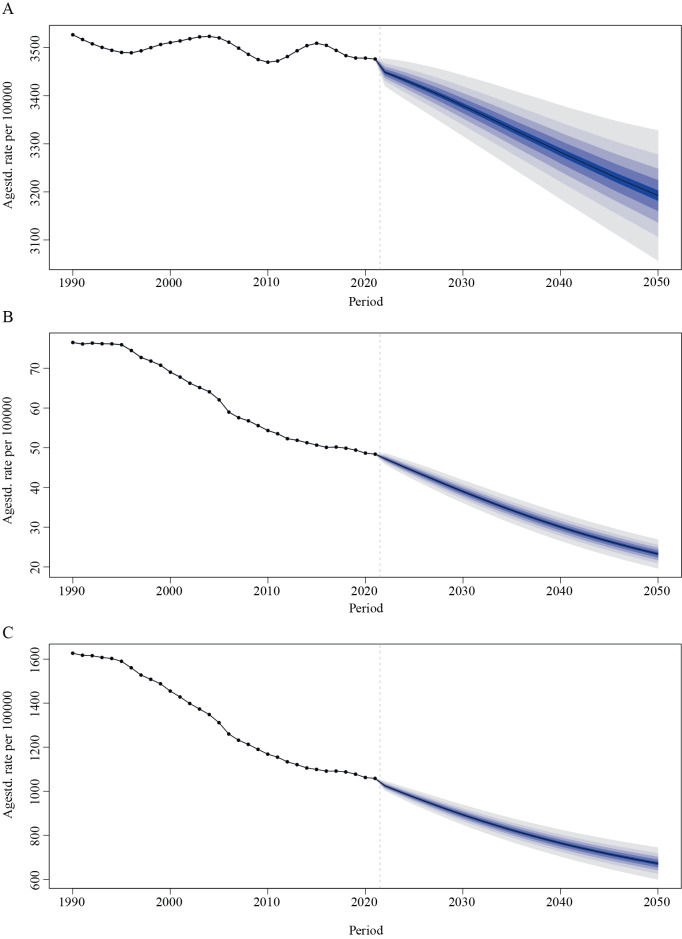
Global predictions of female COPD-related ASPR **(A)**, ASMR **(B)**, and ASDR **(C)** from 2022 to 2050. COPD, chronic obstructive pulmonary disease; ASPR, age-standardized prevalence rate; ASMR, age-standardized mortality rate; ASDR, age-standardized disability-adjusted life years (DALYs) rate.

We employed the BAPC model to predict the number of female COPD patients, deaths, and DALYs in the next 30 years. The results showed that the number of female patients was projected to show an upward trend, while the number of deaths and DALYs was projected to have a declining trend. The number of female COPD patients was expected to rise from 63,558,840.6 in 1990 to 124,365,590.6 in 2050, while the number of female COPD deaths was expected to reduce from 1,378,276.3 in 1990 to 905,885.7 in 2050. The number of DALYs was projected to decline from 29,318,963.2 to 26,179,908.9 (Figure 6 and Table S3). The corresponding ASRs all exhibited a declining trend in the next 30 years (Figure 7 and Table S3).

### Risk factors

**Figure 8:**
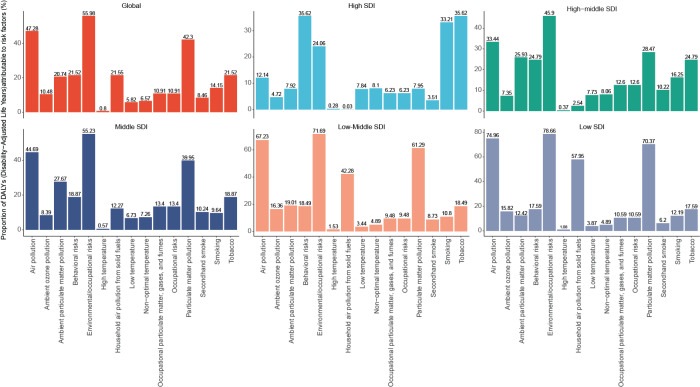
Attributable risk ratios of five-level risk factors for female COPD mortality in 2021 globally **(A)** and across SDI regions **(B, C, D, E, and F)**. COPD, chronic obstructive pulmonary disease; SDI, socio-demographic index (five categories: countries with a high, high-middle, middle, low-middle, or low socio-demographic index).

The risk factors for female COPD-related DALYs in 2021 mainly included two categories: environmental/occupational risks (55.98%) and behavioral risks (21.52%). Specifically, environmental/occupational risks led to 420.3/100,000 DALYs, while behavioral risks led to 161.6/100,000 DALYs. Among the environmental/occupational risks, the main ones were air pollution (47.28%) and particle matter pollution (42.3%). Among them, air pollution led to 354.9/100,000 DALYs, while particle matter pollution led to 317.5/100,000 DALYs. Behavioral risks mainly included smoking (14.15%) and tobacco (21.52%), with smoking causing 106.2/100,000 DALYs, and tobacco causing 161.6/100,000 DALYs. In different SDI regions, the proportion of each risk factor varied. In the high SDI region, the attributable risk ratio of behavioral risks (35.62%) was higher than that of environmental/occupational risks (24.06%). Unlike in the high SDI region, other regions tend to have environmental/occupational risks higher than behavioral risks (Table S4 and Figure 8).

## Discussion

### Principal findings

Based on data from the 2021 GBD study, this research summarized and analyzed the global, regional, and national prevalence, mortality, DALY, and corresponding ASRs of female COPD from 1990 to 2021, and predicted future development trends. In 2021, there were 112.7 million female COPD cases, 1.6 million female COPD deaths, and 34.7 million DALYs worldwide, with corresponding ASRs of 2468.2/100,000, 34.1/100,000, and 750.6/100,000, respectively. The ASRs of female COPD patients were expected to show a declining trend in the next 30 years. To our knowledge, this study is the first to report the global, regional, and national disease burden of female COPD, which can help decision-makers to better understand the situation and formulate specialized prevention and control strategies to reduce the disease burden of female COPD.

The results of this work indicated that although ASPR, ASMR, and ASDR in female COPD patients have reduced in the past 30 years, their numbers are still on the rise, which is consistent with the global trend of COPD changes reported in previous GBD studies [11, 19]. The rise in quantity may be due to global population growth, aging development, and a large female smoking population. The decline in ASRs may be attributed to the implementation of stringent tobacco control policies and advancements in medical technology [20–23].

SDI analysis revealed that the ASMR and ASDR of female COPD patients showed a trend of first rising and then declining with the elevation of SDI, which was in line with the changing trend of COPD in the global population [11]. ASPR of female COPD patients in the low-middle SDI region was at the highest level, ASMR and ASDR gradually increased to the highest levels in recent years. In the process of economic development in the low-middle SDI region, social problems such as tobacco proliferation and environmental pollution have emerged, increasing the risk of female COPD. The insufficient allocation of public health resources in chronic disease management in these areas has further elevated the ASRs of female COPD patients in these areas [24, 25]. In contrast, the ASMR and ASDR in the middle SDI and high-middle SDI regions showed a significant downward trend. Since 2003, when the WHO introduced the Framework Convention on Tobacco Control (FCTC), the prevalence of tobacco use among adult women in these regions has shown a significant decline [20, 26, 27]. In addition, many countries in this region have made tremendous efforts to improve indoor and outdoor environments, such as issuing the *Action Plan for Air Pollution Prevention and Control*, strictly controlling emissions, monitoring pollution concentrations, and implementing economic penalty policies [28] thus improving the environmental issues in such areas. In addition, with the development of the socio-economy, the medical level in these areas continues to improve, public health awareness is raised, and women’s health issues are given more attention. These factors collectively enhance the chronic disease management level of female COPD patients in these areas [8, 29, 30]. Although the prevalence of COPD is relatively high in the high SDI region, its ASMR and ASDR remain at relatively low levels, which may be closely linked to a more comprehensive chronic disease management system, higher early screening and diagnostic capabilities, good medical insurance policies, and higher education levels in high SDI region [29, 31]. It should also be acknowledged that differences in the accessibility to diagnostic procedures and health services across countries may influence the interpretation and generalizability of our findings. In regions with well-developed healthcare systems, patients are more likely to receive timely diagnostic assessments and standardized treatments, which could improve outcomes. Conversely, in countries with limited access to diagnostic tools or constrained healthcare resources, underdiagnosis or delayed treatment may occur, potentially leading to worse prognoses. The factors mentioned above partially explain the variations in the burden of COPD among women across different countries and geographical regions.

We also analyzed the risk factors for female COPD-related DALYs, finding environment and tobacco as one of the factors affecting global female COPD-related DALYs, which is consistent with previous research on the risk factors of global COPD-related DALYs [11, 32]. The growth in female tobacco consumption and their difficulties in quitting smoking [3, 33, 34] lead to an intensified burden of COPD disease in women. Furthermore, exposure to biomass smoke is considered one of the major risk factors for COPD in non-smoking women, especially in developing countries where women often cook or heat in poorly ventilated rooms using fuels such as coal or wood, which can easily produce large amounts of harmful particles.

### Strengths and limitations of this research

A strength of the study is that we have provided up-to-date and comprehensive estimates of levels and trends associated with COPD among females, as well as an analysis of its risk factors at the global, regional, and national levels, between 1990 and 2021. However, some key limitations persist in this research. First, this analysis heavily relies on secondary data from the GBD database, whose accuracy is limited by the availability of national registration data, a large number of undiagnosed female COPD cases, and a lack of information on other risk factors related to female COPD. Second, GBD databases often have delays in data updates. Therefore, the data used may not reflect the actual prevalence trend of COPD in women at present. Third, due to limitations in the database, this project lacks detailed data at the individual level, making it difficult to examine the specific situation and treatment response of patients in depth. These limitations may limit our comprehensive understanding of the etiology and management strategies of COPD in women.

## Conclusions

This investigation reported the latest updated global, regional, and national disease burden and attributable risk factors for female COPD. Although the ASRs of female COPD declined during the study period, the corresponding number of people increased. This reminds us that female COPD is a public health issue that cannot be ignored. In the future, when formulating COPD prevention and treatment strategies, the special characteristics of the female population should be fully considered to reduce their disease burden.

## Data Availability

The data used in this study are publicly available from the GBD database. All data can be accessed through the Global Health Data Exchange at http://ghdx.healthdata.org/gbd-results-tool.

## Additional Files

The additional files for this article can be found as follows:

Supplementary Table 1. Prevalent cases, deaths, and DALYs for COPD among women nationally in 2021, and percentage change in ASRs per 100,000, from 1990 to 2021.

Supplementary Table 2. Numbers and ASRs of prevalence, deaths, and DALYs of COPD among women, by age in 2021.

Supplementary Table 3. Global predictions of female COPD-related numbers and ASRs of prevalence, deaths, and DALYs from 2022 to 2050.

Supplementary Table 4. Attributable risk ratios of five-level risk factors for female COPD mortality in 2021 globally and across SDI regions.
